# Effect of the timing of surgery on the fracture healing process and the expression levels of vascular endothelial growth factor and bone morphogenetic protein-2

**DOI:** 10.3892/etm.2014.1735

**Published:** 2014-05-28

**Authors:** LI-QIANG DONG, HANG YIN, CHANG-XING WANG, WEI-FENG HU

**Affiliations:** Department of Orthopaedics, The Second Affiliated Hospital of Zhejiang Chinese Medical University, Hangzhou, Zhejiang 310005, P.R. China

**Keywords:** fracture healing, vascular endothelial growth factor, bone morphogenetic protein-2, surgery timing

## Abstract

The aim of the present study was to observe the effect of varying the timing of surgery on the fracture healing process and the expression levels of vascular endothelial growth factor (VEGF) and bone morphogenetic protein (BMP)-2 in rats. A total of 192 rats underwent closed femur fracture modelling. The rats underwent open reduction and internal fixation surgery 1, 3, 5, 7, 11 and 14 days subsequent to the fracture occurring. Immunohistochemical staining and analysis of the VEGF and BMP-2 expression levels were simultaneously conducted on bone from the fracture site of the rats on various days. The VEGF and BMP-2 expression levels at the fracture sites were higher and were maintained for a longer period of time in the 7- and 11-day surgery groups than in the other surgery groups and the rats that did not undergo surgery. The 5-day surgery group demonstrated a greater intensity in BMP-2 expression compared with the remaining surgery groups; however, no significant differences were identified between 1-day surgery and non surgery groups. In the 3-day surgery group, the expression levels VEGF and BMP-2 were low at each stage of the fracture-healing process and were lower compared with those observed in the non-surgery group. The timing of the surgical procedures affected the VEGF and BMP-2 expression levels at the fracture sites of the experimental rats and, the optimal time for performing surgery was identified to be within the first two weeks. However, surgery may not be conducive to fracture healing if it is performed within the first few days following fracture.

## Introduction

The recent development of osteosynthesis has revealed a close association between bone fracture treatment and open reduction and internal fixation (ORIF). A previous study demonstrated that the potential for nonunion in patients who have undergone surgical treatment is four times greater than that in patients who have undergone conservative treatment (1). A general surgical incision results in excessive damage to the local blood supply (for example, periosteal stripping), which is a negative factor for fracture healing (2); however, there are few studies that have considered the effect of the timing of surgery. The authors of the present study have observed in the clinic that delayed bone fracture healing or nonunion occurred in certain patients who underwent early surgery, whereas satisfactory healing occurred in patients who received delayed surgery. Thus, the effects of surgical treatment may be hypothesised to be closely associated with the local blood supply and the timing of surgery.

The present study was conducted as a preliminary investigation into the molecular basis for the secondary damage-accelerated fracture-healing phenomenon (3,4) and the optimal time at which to perform surgery following fracture was determined.

## Materials and methods

### Animals

A total of 192 Wistar rats (weight, ~220 g) were used in the present study and provided by the Zhejiang Chinese Medical University Experimental Animal Centre (Hangzhou, China). The rats were subjected to consistent feeding conditions at room temperature (24±2°C) and provided with standard feed (Anritsu mime; Nanjing Science and Technology Co., Ltd., Nanjing, China), with access to food and drinking water *ad libitum*. The rats were subjected to adaptive feeding for more than one week without exception prior to their use in the study. The present study was conducted in strict accordance with the recommendations in the Regulations for the Administration of Affairs Concerning Experimental Animals (Ministry of Science and Technology of China, 1988). The animal use protocol was reviewed and approved by the Institutional Animal Care and Use Committee of Zhejiang Chinese Medical University (Hangzhou, China).

### Closed fracture model

The rats were anaesthetised with 10% sodium pentobarbital (Shanghai Kefeng Chemical Reagent Co., Ltd., Shanghai, China) at a dose of 40 mg/kg via an intraperitoneal injection. The Einhorn fracture modelling method (5,6) for animals was employed to create a closed fracture model in the right femur of the rats. The thumb and index finger were used to assess the fracture site and determine the type of fracture. The middle femur fracture type and short oblique fractures were identified to be common.

### Animal grouping and operation

The 192 closed fracture model rats that complied with the inclusion criteria were randomly divided into seven groups as follows: Group A, no surgery (n=36); group B, surgery one day following fracture (n=36); group C, surgery three days following fracture (n=32); group D, surgery five days following fracture (n=28); group E, surgery seven days following fracture (n=24); group F, surgery 11 days following fracture (n=20); and group G, surgery 14 days following fracture (n=16).

In group A, four modelled rats were sacrificed by cervical dislocation and observed at 8 h and 3, 5, 7, 11, 14, 21, 35 and 49 days following fracture. ORIF was performed on the rats in groups B, C, D, E, F and G at 1, 3, 5, 7, 11 and 14 days after fracture, respectively. The surgery was conducted as follows: The rats were anaesthetised with 10% sodium pentobarbital at a dose of 40 mg/kg by intraperitoneal injection. They were placed in the prone position and their right hind limbs were prepared for surgery. Following a routine disinfection, a posterolateral incision was made on the right femur, and the subcutaneous tissue and right rear muscle were separated to reveal the femur fracture. The periosteum was protected and the fracture was reduced. An incision was made on the knee joint capsule to expose the lateral condyle of the femur with an intercondylar diameter of l.5 mm. A Kirschner wire was inserted to fix the fracture fragments, and the incision was sutured and bandaged with gauze. The rats were administered penicillin injections for three days to prevent the occurrence of postoperative infections.

Four rats from each group (B, C, D, E, F and G) were sacrificed by breaking of the neck and were observed at different time points. For group B, the time points were 8 h after surgery, and 3, 5, 7, 11, 14, 21, 35 and 49 days following fracture. For group C, the time points were 3 (8 h after surgery), 5, 7, 11, 14, 21, 35 and 49 days following fracture. For group D, the time points were 5 (8 h after surgery), 7, 11, 14, 21, 35 and 49 days following fracture. For group E, the time points were 7 (8 h after surgery), 11, 14, 21, 35 and 49 days following fracture. For group F, the time points were 11 (8 h after surgery), 14, 21, 35 and 49 d following fracture. For group G, the time points were 14 (8 h after surgery), 21, 35 and 49 days following fracture.

After the rats were sacrificed, the 1 cm bone tissue with intact periosteum and bone callus at fracture region was taken.

### Immunohistochemical staining

Immunohistochemical staining for VEGF and BMP-2 was performed. The bone tissue was fixed in 10% neutral buffered formalin (Guangzhou Wexis Biotech Co., Ltd., Guangzhou, China), washed with distilled water, placed in 5% ethylene diamine tetraacetic acid solution (Beijing Century Aoke Biotech Co., Ltd., Beijing, China) and decalcified for 10–15 days. Following decalcification, the specimens were flushed for 24 h, dehydrated and embedded in paraffin in 5-μm thick serial sections. The samples were immunohistochemically stained to detect the expression of VEGF and BMP-2 (streptavidin biotin complex kit reagent; Wuhan Boster Biological Technology, Ltd., Wuhan, China) using the diaminobenzidine chromogenic method; a brown stain indicated a positive result. The VEGF and BMP-2 content in the callus was determined using an internet printing protocol computer with a colour image analysis system (Image-Pro Plus 6.0; Media Cybernetics Inc., Bethesda, MD, USA). Under a microscope (magnification, ×400; B×50; Olympus Corp., Tokyo, Japan), five areas were randomly selected for each specimen to perform the expression counts, and the count values of the VEGF and BMP-2 expression were taken as the measured indicators (7,8); the results were statistically analysed.

### Statistical analysis

SPSS software, version 13.0 (SPSS Inc., Chicago, IL, USA) was used to perform the statistical analysis. The data are expressed as means ± standard deviation and differences between the groups were compared using Student’s t-test. P<0.05 was considered to indicate a statistically significant result.

## Results

### VEGF-positive cells

At 14 days following fracture, the VEGF expression in groups A, B, C and D peaked and gradually decreased thereafter. In groups E, F and G, the VEGF expression continued to increase between 14 and 21 days following fracture and peaked at day 21, followed by a gradual decline. However, the peak values for groups F and G demonstrated no significant difference (P>0.05). At day 35 for each group VEGF expression was marginally elevated; however, the VEGF expression levels in groups E, F and G were highest.

**Figure 1:**
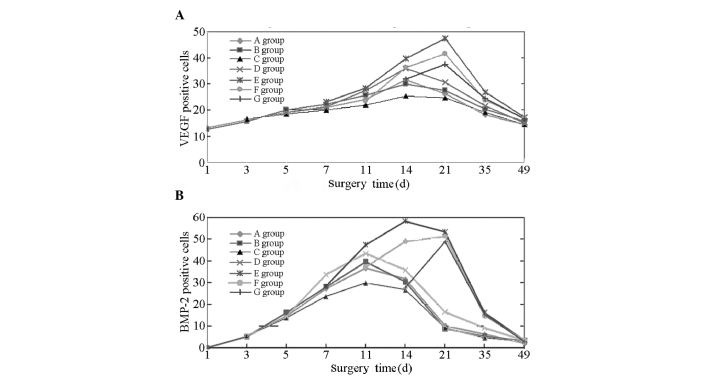
Quantity-change curves of (A) VEGF and (B) BMP-2-positive stained cells at various times following surgery conducted at different times after fracture. VEGF, vascular endothelial growth factor; BMP-2, bone morphogenetic protein-2.

At 14 days, the results of the comparison indicated that the VEGF expression levels decreased in the order: E, F and D > A, B and G > C. No significant differences (P>0.05) were observed in VEGF expression between groups A, B and G. Significant differences were observed between groups E, F and D; A, B and G; and C (P<0.05 or P<0.01). At 21 days, the results of the comparison demonstrated that VEGF expression decreased in the order: E, F and G > D > A, B and C. No significant differences were observed between groups A, B and C (P>0.05). Significant differences were observed between groups E, F and G; D; and A, B and C (P<0.05 or P<0.01). At 35 days, the results of the comparison show that the VEGF expression decreased in the order: E > F and G > A, B, C and D. No significant difference was observed between groups A, B, C and D (P>0.05). Significant differences were observed between groups E; F and G; and A, B, C and D (P<0.05 or P<0.01). At 49 days, the fractures exhibited gradual healing. A marginal quantity of VEGF expression remained in each group and the differences observed between the groups were not identified to be statistically significant (P>0.05; Fig. 1A and Table I).

### BMP-2-positive cells

After 11 days, the BMP-2 expression levels in groups A, B, C and D peaked and gradually decreased thereafter. For groups E and F, the curve of BMP-2 expression continued to increase 14 days following fracture. Furthermore, BMP-2 expression peaked in group E on day 14 and began to decline after day 21. BMP-2 expression peaked in groups F and G at 21 days and then gradually decreased. At 35 days in each group, BMP-2 expression was detected; however, the expression levels were highest in groups E, F and G. At day 49, the fractures exhibited gradual healing and only a marginal quantity of BMP-2 expression was evident in each group.

On day 1, the expression levels in groups A and B were not significantly different. On day 3, a low expression level of BMP-2 was observed in groups A, B and C, with no significant difference identified between the groups (P>0.05). On day 5, the expression levels in groups A, B, C and D increased further, with no significant differences identified between the groups (P>0.05). By day 7, the expression levels in groups A, B, C, D and E were increased significantly, in the order: D >A, B and E > C. A comparison between groups A, B and E indicated no significant differences (P>0.05). The differences among the remaining groups were identified to be statistically significant (P<0.05 or P<0.01). On day 11, the results of the comparison demonstrated that BMP-2 expression was increased in the order: E > D >A, B and F > C. The differences between groups A, B and F were not identified to be statistically significant (P>0.05); however, the differences were statistically significant for the remaining groups (P<0.05 or P<0.01). At day 14, a comparison of BMP-2 expression revealed that: E > F > D > A, B and G > C. The differences among groups A, B and G were not identified to be statistically significant (P>0.05); however, the differences were statistically significant among the remaining groups (P<0.05 or P<0.01). At day 21, the comparison of BMP-2 shows the following: E, F and G > D > A, B and C. The value in group E remained the highest. Comparisons between two groups (E and F; F and G) indicated no statistically significant differences (P>0.05). Furthermore, no significant difference was observed between groups A, B and C (P>0.05); however, the differences among the remaining groups were statistically significant (all P<0.05 or P<0.01). At day 35, the expression levels observed in each group decreased and those in groups E, F and G fell markedly; however, they were greater than those in the other groups; the expression levels in groups A, B and C were the lowest. A comparison of BMP-2 expression levels indicated the following: E, F and G > D >A, B and C. The differences between groups A, B and C were not identified to be statistically significant (P>0.05); however, those among the remaining groups were statistically significant (P<0.05 or P<0.01). On day 49, a marginal quantity of BMP-2 expression remained in each group and the differences between the groups were not identified to be significant (P>0.05; Fig. 1B and Table II).

## Discussion

The histological healing process may be accompanied by a complex biological regulatory mechanism. A large number of cytokines in the various stages of fracture healing have different functions. Angiogenesis, which is the premise of fracture repair, is the restoration of blood flow and VEGF is the predominant regulator of this process. Furthermore, binding of the vascular endothelial cell membrane receptor specifically promotes vascular endothelial cell proliferation and angiogenesis (9). The function of oxygen in angiogenesis is significant in the fracture segment areas as it provides nutrients for metabolic waste transport. In addition, it provides a favourable microenvironment for local bone regeneration and metabolism. Spector *et al* (10) found that VEGF *in vitro* did not result in osteoblast proliferation, but led to migration and osteoblast differentiation. Furthermore, the desired concentration of VEGF which leads to osteoblast migration and differentiation is 100 times lower than that of BMP-2. Another study showed that VEGF in original osteoblasts has a chemotactic effect on bone formation and reconstruction in the endochondral bone, particularly in the coupling of bone formation in the cartilage absorption process (11). Connoly (12) indicated that fracture healing relied on the revascularization process and the evaluation of fracture healing was also entirely dependent on the revascularization procedures. BMP has a major function in the differentiation of original bone cells and is significant in promoting osteogenesis (13–15). BMP is one of the cell factors of the transforming growth factor 2β superfamily, which is a group of multifunctional cytokines that induce the migration, proliferation and differentiation of mesenchymal cells resulting in cartilage and bone formation. The majority of studies have focused on the osteogenic effect of BMP-2. *In vivo* and *in vitro* experiments have shown that BMP-2 is able to induce osteoblast differentiation and bone formation, and is a potent cytokine (16–18). Furthermore, the presence of BMP-2 has been observed in various stages of bone formation (19). Bouletreau *et al* (20) confirmed that hypoxia or the use of exogenous VEGF promotes BMP-2 mRNA and protein expression after 24–48 h of the fracture-healing process. This finding indicates that vascular endothelial cells have a function in angiogenesis and BMP-2 expression results in osteogenesis in the fractured region. Therefore, VEGF and BMP-2 were used as indicators in the present study to observe the different fracture healing effects following surgery conducted at various time points after fracture.

The results indicated that the VEGF and BMP-2 expression levels in groups E, F and G were significantly higher and remained high for longer than the levels in groups A, B, C and D. In groups E, F and G, the strength of the expression levels were in the following order: E > F > G. Group D exhibited relatively low expression levels, which were lower than those observed in groups E, F and G, and marginally higher than those in groups A and B; group C demonstrated the lowest expression levels. The VEGF and BMP-2 expression levels during the fracture healing process in each group were in the following order: E > F > G > D > B = A > C. Thus, surgery during the different stages of fracture healing in rats was shown to affect the VEGF and BMP-2 expression levels and the duration of the expression. Although the timing of surgery showed no beneficial effects, the 7-day surgery group demonstrated the highest VEGF and BMP-2 expression levels during fracture healing, and the effects endured for longer. In addition, in the 11-day surgery group, the VEGF and BMP-2 expression levels were promoted and the effects endured for a long time. The expression levels of the 14-day surgery group were comparable with those in the 7- and 11-day surgery groups. In the 5-day surgery group, the expression intensity was higher than that observed in the group with natural healing and no surgery. There was no significant difference between the 1-day surgery group and non-surgery group, and no significant effect on the VEGF and BMP-2 expression levels at fracture was observed. The three-day surgery group showed low levels of VEGF and BMP-2 at all stages of fracture healing and these levels were lower than those of the non-surgery group.

VEGF was positively expressed during the entire process of fracture healing. The VEGF expression became visible on the first day following fracture, whereas BMP-2 expression became visible in the positively stained cells on day 3. The findings of the present study were consistent with the study by Bouletreau *et al* (20); VEGF may have induced or promoted the expression of BMP-2. After 7–28 days, the peak in VEGF expression was accompanied by a peak in angiogenesis; thus, it was hypothesized that VEGF and visible vascular reconstruction in the fracture were closely associated. Previous studies (7,21,22) have indicated that fractures caused by trauma result in the partial interruption of blood flow and weak VEGF expression in the following week. However, the process of fracture repair accelerates the growth of granulation tissue, reduces the blood supply and decreases the partial pressure of oxygen. In addition, hypoxia strongly induces VEGF expression. In the present study, 2–3 weeks after fracture, VEGF mRNA expression was observed to peak, which indicated that the synthesis of cartilage cells was occurring. In the endochondral ossification process, VEGF is involved in the coordination of cartilage cells for bone cell transformation (7,21,22). In the present study, surgery conducted several days after the fracture procedure may have aggravated the trauma and damaged the periosteum of the blood vessels, resulting in an interruption of the increased blood flow and reducing the VEGF expression. In the initial two weeks, granulation growth around the fracture was apparent and periosteal thickening occurred. Although the periosteum was completely cut during surgery, less damage was observed in blood flow of the fracture end. Furthermore, the time period of inflammation was extended and local capillary generation was promoted, which further strengthened the local blood supply. Thus, surgery contributed to fracture healing, which was closely associated with VEGF secretion. In groups A and B, BMP-2 expression was stronger in the initial stage of the fracture healing process (5–14 days following fracture) and gradually decreased in the later stage of fracture healing (21–49 days following fracture). The other groups demonstrated different BMP-2 expression levels due to the varied timing of the surgery. In the 7- and 14-day surgery groups, the BMP-2 expression was observed to be more intense and endured for longer; the BMP-2 expression was most apparent in the 7-day surgery group. The results indicated that performing a surgical procedure 7 days after the fracture was optimal for promoting callus BMP-2 release and regeneration.

In conclusion, post-fracture surgical procedures may affect the VEGF and BMP-2 expression levels in rats. Surgery may lead to the acceleration of secondary damage. The optimal timing of surgery was identified to be within one to two weeks of fracture. Conducting a surgical procedure several days after fracture may not be conducive to fracture healing.

## Figures and Tables

**Table I:** VEGF-positive immunohistochemical staining cell counts at various times following surgery conducted at different times after fracture (means ± standard deviation; n=4).

Group	Day 1	Day 3	Day 5	Day 7	Day 11	Day 14	Day 21	Day 35	Day 49
A	13.0±1.52	16.2±2.04	19.0±2.27	21.0±2.86	24.2±2.03^c^,^e^	31.5±1.88^b^,^c^,^f^,^g^	26.2±3.01^f^,^h^,^j^	18.1±2.91^f^,^g^,^i^	14.5±2.40
B	12.6±1.70	15.5±2.30	20.1±2.30	22.1±2.15	25.5±1.95	30.0±2.67^a^,^c^,^f^,^g^	27.5±2.71^f^,^h^,^j^	20.3±2.43^e^	15.6±2.47
C	-	16.5±1.40	18.3±1.55	20.1±2.24	22.0±2.34^d^,^f^	25.2±2.50^d^,^f^,^h^	24.6±3.03^f^,^h^,^j^	19.2±2.17^f^,^g^,^i^	14.4±2.44
D	-	-	19.0±1.97	21.2±1.77	27.5±1.72	35.8±2.78^b^	30.5±2.97^a^,^f^,^h^,^i^	21.5±2.59^e^	15.0±2.33
E	-	-	-	23.0±2.45	28.5±2.50	39.8±3.92^b^	47.6±4.10	26.9±2.66	17.2±2.59
F	-	-	-	-	23.5±2.57^c^,^e^	36.2±3.22^b^	41.5±3.80	23.8±2.59	16.8±2.45
G	-	-	-	-	-	32.0±3.29^a^,^e^	37.4±3.00^f^	24.5±2.83	16.6±2.16

a P<0.05 and

b P<0.01 compared with group C;

c P<0.05 and

d P<0.01 compared with group D;

e P<0.05 and

f P<0.01 compared with group E;

g P<0.05 and

h P<0.01 compared with group F;

i P<0.05 and

j P<0.01 compared with group G.

**Table II:** BMP-2-positive immunohistochemical staining cell counts at various times following surgery conducted at different times after fracture (means ± standard deviation; n=4).

Group	Day 1	Day 3	Day 5	Day 7	Day 11	Day 14	Day 21	Day 35	Day 49
A	0	5.2±0.62	14.6±1.10	27.2±1.12^a^,^d^	36.5±1.88^b^,^d^,^f^	31.5±1.57^b^,^d^,^f^,^h^	10.2±1.10^d^,^f^,^h^,^j^	6.2±1.20^a^,^f^,^h^,^j^	3.2±0.43
B	0	5.1±0.38	16.2±1.72	28.1±1.00^b^,^d^	39.4±2.43^b^,^c^,^f^	30.2±1.66^a^,^d^,^f^,^h^	8.9±1.50^d^,^f^,^h^,^j^	5.3±1.10^d^,^f^,^h^,^j^	3.2±0.52
C	-	5.4±0.59	13.8±1.93	23.5±1.72	29.8±1.54	26.6±1.44	9.1±1.16	4.5±1.28	2.8±0.59
D	-	-	14.5±1.41	33.6±1.75^b^	43.2±1.36^b^,^f^,^h^	35.7±1.25^b^,^f^,^h^	16.5±1.44^b^,^f^,^h^,^j^	9.2±1.30^b^	3.5±0.89
E	-	-	-	28.4±1.30^b^,^d^	47.3±1.72^b^,^h^	58.2±2.38^b^,^d^,^h^	53.2±2.54^b^,^i^	16.1±1.47^b^^d^	3.3±0.81
F	-	-	-	-	37.1±1.31^b^	48.8±2.11	51.1±2.16^b^	14.7±1.37^b^^d^	3.6±0.71
G	-	-	-	-	-	29.6±2.07^d^,^f^	48.7±2.46^b^	15.3±0.96^b^^d^	2.9±0.99

a P<0.05 and

b P<0.01 compared with group C;

c P<0.05 and

d P<0.01 compared with group D;

e P<0.05 and

f P<0.01 compared with group E;

g P<0.05 and

h P<0.01 compared with group F;

i P<0.05 and

j P<0.01 compared with group G.

## References

[1] Borrelli J, Prickett WD, Ricci WM (2003). Treatment of nonunions and osseous defects with bone graft and calcium sulfate. Clin Orthop Relat Res.

[2] Wolf JM, Athwal GS, Shin AY, Dennison DG (2010). Acute trauma to the upper extremity: what to do and when to do it. Instr Course Lect.

[3] Cornell CN, Lane JM (1992). Newest factors in fracture healing. Clin Orthop Relat Res.

[4] Hulth A (1989). Current concepts of fracture healing. Clin Orthop Relat Res.

[5] Makino T, Hak DJ, Hazelwood SJ, Curtiss S, Reddi AH (2005). Prevention of atrophic nonunion development by recombinant human bone morphogenetic protein-7. J Orthop Res.

[6] Shefelbine SJ, Simon U, Claes L (2005). Prediction of fracture callus mechanical properties using micro-CT images and voxel-based finite element analysis. Bone.

[7] Kaspar D, Neidlinger-Wilke C, Holbein O, Claes L, Ignatius A (2003). Mitogens are increased in systemic circulation during bone callus healing. J Orthop Res.

[8] Hughes-Fulford M, Li CF (2011). The role of FGF-2 and BMP-2 in regulation of gene induction, cell proliferation and mineralization. J Orthop Surg Res.

[9] Millauer B, Wizigmann-Voos S, Schnürch H (1993). High affinity VEGF binding and developmental expression suggest Flk-1 as a major regulator of vasculogenesis and angiogenesis. Cell.

[10] Spector JA, Mehrara BJ, Greenwald JA (2001). Osteoblast expression of vascular endothelial growth factor is modulated by the extracellular microenvironment. Am J Physiol Cell Physiol.

[11] Mayr-Wohlfart U, Waltenberger J, Hausser H (2002). Vascular endothelial growth factor stimulates chemotactic migration of primary human osteoblasts. Bone.

[12] Connolly JF (1995). Injectable bone marrow preparations to stimulate osteogenic repair. Clin Orthop Relat Res.

[13] Roberts AB, Sporn MB, Assoian RK (1986). Transforming growth factor type beta: rapid induction of fibrosis and angiogenesis in vivo and stimulation of collagen formation in vitro. Proc Natl Acad Sci U S A.

[14] Song I, Kim BS, Kim CS, Im GI (2011). Effects of BMP-2 and vitamin D3 on the osteogenic differentiation of adipose stem cells. Biochem Biophys Res Commun.

[15] Huang H, Song TJ, Li X (2009). BMP signaling pathway is required for commitment of C3H10T1/2 pluripotent stem cells to the adipocyte lineage. Proc Natl Acad Sci U S A.

[16] Chen D, Harris MA, Rossini G (1997). Bone morphogenetic protein 2 (BMP-2) enhances BMP-3, BMP-4, and bone cell differentiation marker gene expression during the induction of mineralized bone matrix formation in cultures of fetal rat calvarial osteoblasts. Calcif Tissue Int.

[17] Barradas AM, Fernandes HA, Groen N (2012). A calcium-induced signaling cascade leading to osteogenic differentiation of human bone marrow-derived mesenchymal stromal cells. Biomaterials.

[18] Bramono DS, Murali S, Rai B (2012). Bone marrow-derived heparan sulfate potentiates the osteogenic activity of bone morphogenetic protein-2 (BMP-2). Bone.

[19] Onishi T, Ishidou Y, Nagamine T (1998). Distinct and overlapping patterns of localization of bone morphogenetic protein (BMP) family members and a BMP type II receptor during fracture healing in rats. Bone.

[20] Bouletreau PJ, Warren SM, Spector JA (2002). Hypoxia and VEGF up-regulate BMP-2 mRNA and protein expression in microvascular endothelial cells: implications for fracture healing. Plastic Reconstr Surg.

[21] Park SH, O’Connor KM, McKellop H (2003). Interaction between active motion and exogenous transforming growth factor Beta during tibial fracture repair. J Orthop Trauma.

[22] Szczesny G (2002). Molecular aspects of bone healing and remodeling. Pol J Pathol.

